# Molecular and hematological studies in a cohort of beta zero South East Asia deletion (β°-thal SEA) from Malaysian perspective

**DOI:** 10.3389/fped.2022.974496

**Published:** 2022-11-30

**Authors:** Norafiza Mohd Yasin, Faidatul Syazlin Abdul Hamid, Syahzuwan Hassan, Aziee Sudin, Haiyuni Yassim, Ermi Neiza Mohd Sahid, Yuslina Mat Yusoff, Ezalia Esa, Mohamed Saleem

**Affiliations:** ^1^ Hematology Unit, Cancer Research Centre, Institute for Medical Research, Ministry of Health (Malaysia), Kuala Lumpur, Malaysia; ^2^ Advanced Genomics Sdn Bhd, Petaling Jaya, Malaysia

**Keywords:** beta-thalassaemia, HPFH, deltabeta thalassaemia, deletional mutation, β°-thal SEA, β° deletion

## Abstract

**Abstract:**

We report the haematological parameters and molecular characterization of beta zero (β°) South East Asia (SEA) deletion in the *HBB* gene cluster with unusually high levels of Hb F compared to a classical heterozygous beta zero (β°)-thalassaemia.

**Methods:**

Retrospective study on 17 cases of (β°) South East Asia (SEA) deletion from 2016 to 2019 referred to Institute for Medical Research were conducted. The clinical information and haematological profiles were evaluated. The mutation was analyzed, and the results were compared with other β°-thalassaemia groups. For *HBB* gene genotyping, all the cases were subjected for multiplex gap-PCR, 5 cases were subjected for *HBB* gene sequencing for exclusion of compound heterozygous with other beta variants. Co-inheritance of α-thalassaemia were determined using multiplex gap-PCR and multiplex ARMS-PCR.

**Results:**

Seventeen cases were positive for β°-thal SEA deletion. Fifteen cases were heterozygous and two were compound heterozygous for β°-thal SEA deletion. The results were compared with 182 cases of various heterozygous β° deletions and mutations. The mean Hb for heterozygous β°-thal SEA deletion (13.44 ± 1.45 g/dl) was normal and significantly higher than heterozygous IVS 1-1 and Codon 41/42 (*post hoc* test, *p* < 0.05). The medians for the MCV and MCH of β°-thal SEA deletion were significantly higher than for all heterozygote β°-thalassaemia traits (Mann Whitney test, *p* < 0.05). Patients with β°-thal SEA deletion had elevated levels of Hb A2 consistent with β-thalassaemia traits, with Hb F levels consistent with HPFH or δβ-thalassaemia carriers. The median for Hb A2 was 4.00 + 1.00%, similar to that observed in other β°-thalassaemia groups except for IVS 1-1 mutation (median 5.30 + 0.45%) and β°-Filipino (∼45 kb deletion) deletion (median 6.00 + 0.58). Interestingly, we found that Hb F levels for β°-thal SEA deletion were statistically higher than other β°-thalassaemia mutations (median 19.00 + 5.50%, *p* < 0.05), except for the β°-thal 3.5 kb deletion group.

**Conclusion:**

We conclude that β°-thal SEA deletion has a unique haematological parameters of beta zero thalassaemia trait. We affirm to classifying this deletion as SEA-HPFH based on previous studies considering the phenotype features rather than the molecular defect of β°-thal SEA deletion, as this will make it easier to offer genetic counselling to affected individuals.

## Introduction

β-thalassemia is a condition resulting from a quantitative reduced rate in β-globin chain synthesis from the *HBB* (β-globin) gene. More than 900 β-globin gene mutations have been characterised, occurring in a wide range of ethnic groups (http://globin.cse.psu.edu/hbvar/menu.html, 2020). The molecular basis of β-thalassemia mutations is divided into two phenotypic groups, β^+^ and β°-thalassemia that reflect the impaired level of β-globin chain synthesis. The β^+^-thalassemia mutations result in a quantitative reduction in the production of the β-globin chain, while β°-thalassemia is marked by the absence of β-globin chain synthesis. Molecular defects in β°-thalassemia could either be a point mutation in the *HBB* gene that completely disrupts its expression or a rare gene deletion that causes absent synthesis of the β-globin chain. Deletions in β-thalassemia represent about 5%–10% of the mutations in the β-globin gene cluster (1, 2).

In the Southeast Asian population, at least 12 types of deletions in the β-globin gene cluster have been described (3). Deletions involving the β-globin gene can be classified into two groups: a group of deletions that are restricted to the HBB gene and another group of larger deletions affecting the β locus control region (LCR) upstream, with or without *HBB* gene involvement (4). More than 150 deletions involving the β-globin gene cluster have been described (5).

Significant numbers of large deletions have been discovered, characterized and reported using various conventional techniques such as gap polymerase chain reaction (Gap-PCR), southern blot analysis, fluorescent *in situ* hybridisation (FISH) and gene mapping analysis by restriction endonucleases (2, 6, 7). These techniques and the analysis involved laborious methods that focus on targeted mutations which are unable to detect novel deletions (2). More sensitive and rapid methods have been described including Multiplex Ligation-dependent Probe Amplification (MLPA) and direct DNA sequencing for deletion characterization (1, 2).

**Figure 1:**
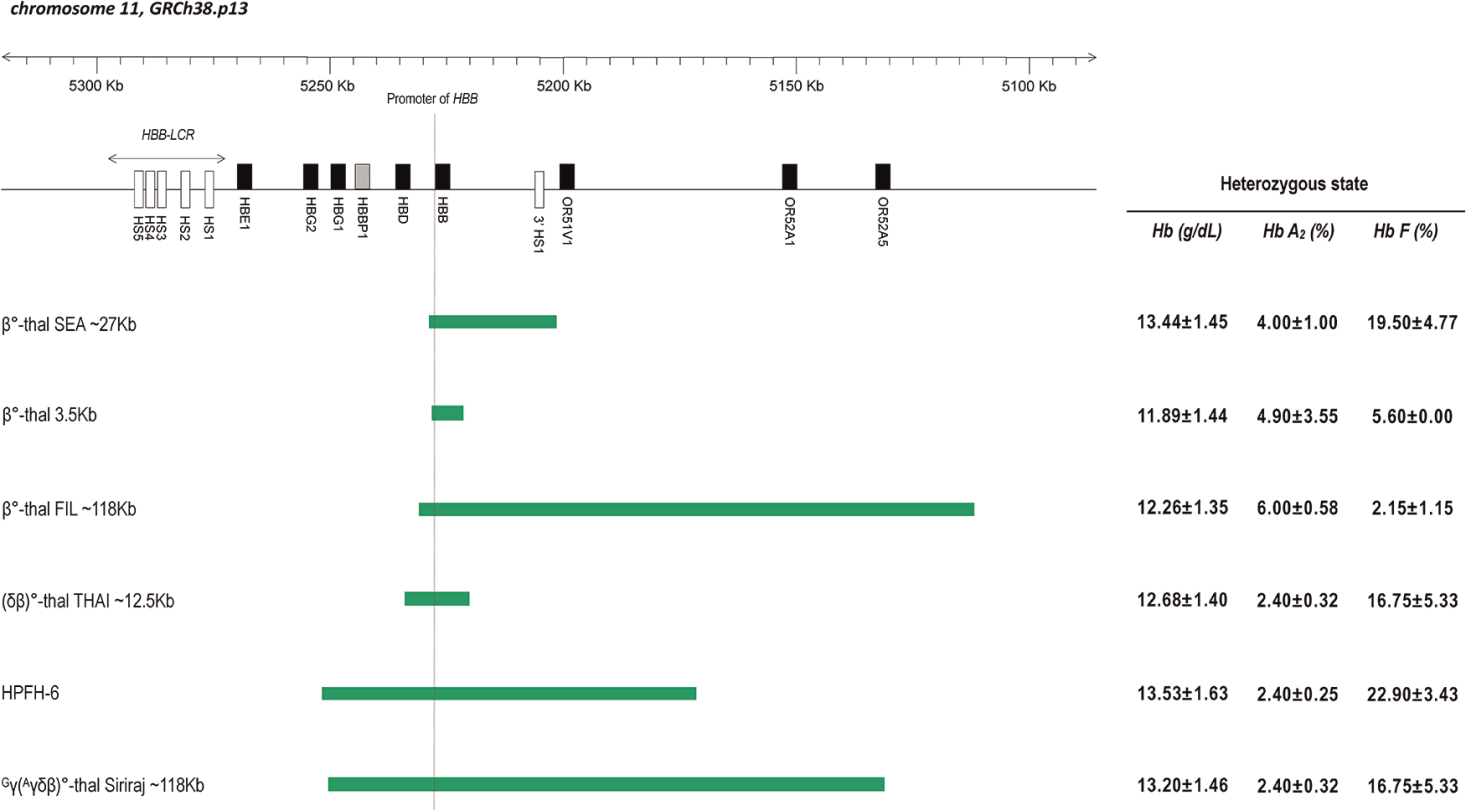
Scheme of β-globin gene cluster in chromosome 11 based on the coordinate from genome reference consortium human build 38 patch release 13 (GRCh38.p13). Genes are indicated by black filled rectangular; pseudogene is indicated by shaded rectangular; DNA Hypersensitive Sites (HS) are indicated by empty rectangular. The dashed line indicates the promoter area of the β-globin gene (*HBB* gene). The region of various deletion types is shown by the green bar. On the right side is the mean value of Hb, Hb A_2_ and Hb F levels found in the heterozygous state for each type of deletion.

The presumptive diagnosis of β-thalassemia is based on the detection of an increase in Hb A_2_ percentage *via* capillary electrophoresis (CE) or high-performance liquid chromatography (HPLC). The percentage of Hb A_2_ is dependent on the precise mutation present (8); in most cases of heterozygous β° or severe β^+^-thalassemia, the Hb A_2_ percentage is between 4% and 5%, whereas for heterozygous mild β^+^-thalassemia this value usually ranges between 2.6% and 4.4% of Hb A_2_ (9). Higher percentages of Hb A_2_ are seen with the β-thalassemia trait due to the deletion of 5’ region of the β-globin gene (6). In β-thalassemia carrier, Hb F levels are classically elevated by between 2% and 7% of total hemoglobin, but the level is not essential for the diagnosis of β-thalassemia (8). The level of Hb F also influenced by the nature of the mutation and its location in the β-globin cluster (10, 11). It has further been shown that the deletion of 5’ region of the β-globin gene will cause elevations of Hb F (Figure 1) (8). A map of few deletions affecting the 5’ β-globin gene region shown in Figure 1.

With the increasing incidence of β-thalassemia in Malaysia, most studies focus on screening of the *HBB* gene for point mutations to establish β-thalassemia, but large deletions are not routinely addressed affecting the sensitivity of the testing. Institute for Medical Research (IMR), Malaysia is one of the main referral centre for the genotyping analysis of hemoglobin disorders in Malaysia and we analyze both point mutations and deletions in the β-globin gene complex to make a comprehensive molecular diagnosis of β-thalassemia.

The main goal of this work is to describe the molecular and haematological parameters in a cohort β° SEA deletion (HGVS nomenclature: NG_000007.3:g.68558_95969del) that has unusually high levels of Hb F for a beta thalassaemia group. We also compared this variants with other forms of beta zero thalassemia, delta beta thalassemia and HPFH in heterozygous form. The possible mechanisms of the enhanced γ-gene expression were discussed.

## Materials and methods

### Subject recruitment

This was a retrospective study on DNA analysis data of beta thalassaemia cases kept from January 2016 to December 2019. The samples were referred from other hospitals to our institution for confirmation of presumptive diagnosis determined from thalassaemia screening tests. We retrieved 17 cases who were genotype as β°-thal SEA ∼27 kb deletion from 2016 to 2019. The mutation was analyzed and the results were compared with 182 cases of other heterozygous β°-thalassemia groups from our database namely [β°-thal FIL ∼45 kb deletion, IVS 1-1 (G > T) (β°) (HBB:c.92 + 1G > T), β°-thal 3.5 kb deletion, Codon 41/42 (-TTCT) (β°) (HBB:c.126_129delCTTT)], (δβ)° group [(δβ)°-thal THAI ∼12.5 kb deletion] and the HPFH group [HPFH-6 and ^G^γ(^A^γδβ)°-thal Siriraj ∼118 kb deletion] for better case discussion and elucidation of the mutations. All the samples used for comparison had completed alpha and beta genotypes. The statistical analysis exclusively involved samples from participants more than 14 years old and did not include any pregnancy individuals. The mutations selection for comparison purposes is done based on the types of genetic lesion and the clinical phenotypes. Beta gap-PCR was performed for all samples, demographic and haematological data, including complete blood count and red blood cell indices (MCV, MCH and RDW) and Hb A_2_ and Hb F levels from either CE or HPLC, were retrieved from the clinical information sheets.

### Thalassaemia screening tests and DNA analysis

The thalassaemia screening tests consisted of full blood count (FBC) and Hb analysis. The FBC was done using automated haematology analyser. The Hb analysis was performed according to a set of tests, i.e., peripheral blood film, CE (SEBIA, France), HPLC (Bio-Rad Laboratories, USA) and Hb electrophoresis (SEBIA, France). Based on Hb analysis findings, majority of the cases were presumptively reported as heterozygous beta thalassaemia, however in view of higher HbF level than expected in classical heterozygous beta thalassaemia cases, a compound heterozygous of beta thalassaemia and deltabeta (δβ) or Hereditary Persistence Foetal Haemoglobin (HPFH) were suspected.

The definitive diagnosis was made through by DNA analysis. Genomic DNA of the cases was extracted from peripheral blood sample using a QIAsymphony DSP DNA Kit (Qiagen GmbH, Hilden, Germany) according to the manufacturer's instructions. All the samples were subjected to β-MARMS and β GAP-PCR. For detection of eight β-globin cluster deletions, we used a simple molecular technique of multiplex gap-PCR assay described by (3). β-thalassemia deletion listed in the multiplex gap-PCR panel were β°-thal SEA ∼27 kb deletion, β°-thal FIL ∼45 kb deletion, β°-thal 3.5 kb deletion, HPFH-6 deletion, ^G^γ(^A^γδβ)°-thal Siriraj ∼118 kb deletion, (δβ)°-thal THAI ∼12.5 kb deletion and Hb Lepore (3). For exclusion of compound heterozygous cases, the samples were further analyze by β-MARMS PCR to rule out cap + 1 (A > C) (β^+^) (HBB:c.-50A > C), codon 19 (AAC > AGC) Hb Malay (β^+^) (HBB:c.59A > G), IVS 1-5 (G > C) (β^+^) (HBB:c.92 + 5G > C), Codon 41/42 (-TTCT) (β°) (HBB:c.126_129delCTTT) and Poly A (AATAAA > AATAGA) (β^+^) (HBB:c.*112A > G) (12). Five cases with β°-thal SEA deletion were randomly selected for β-sequencing to exclude other compound mutations that could lead to higher Hb F level (supplementary data). In addition, multiplex gap-PCR and multiplex ARMS-PCR for HBA1 and HBA2 gene were performed in all 17 cases with β°-thal SEA ∼27 kb deletion and comparison samples to detect two single gene deletions α-^3.7^ and -α^4.2^, and three double gene deletions, --^SEA^, --^MED^ and -(α)^20.5^. The multiplex ARMS-PCR was used to detect the non-deletional α-thalassaemia such as Hb Constant Spring, Hb Quong Sze, Hb Adana, initiation codon mutation (ATG→A–G), codon 30 mutation (ΔGAC) and codon 35 mutation (TCC→CCC). In view of all cases of β°-thal SEA deletion had normal alpha genotype, we exclude the comparison samples with co-inheritance alpha mutation or deletion and incomplete alpha genotype from the statistical analysis.

### Statistical analysis

The demographic data including the states and ethnicity of β°-thal SEA deletion were analyzed by descriptive analysis. All statistical analyzed were performed using the SPSS software (Ver. 22, SPSS Inc., Chicago, USA). Haematological parameters and Hb profiles were compared between all heterozygous cases of β°-thalassemia, HPFH group [HPFH-6 and ^G^γ(^A^γδβ)°-thal Siriraj ∼118 kb deletion] and (δβ)° group [(δβ)°-thal THAI ∼12.5 kb deletion] using one-way analysis of variance (ANOVA) and Kruskal Wallis test. *Post hoc* and Man Whitney test comparisons were performed to evaluate pairwise differences among the groups. Means were reported with standard deviation (SD) and medians with interquartile range (IQR). A *p*-value < 0.05 was considered statistically significant. Data and results were presented in the form of figures and table.

### Ethical approval

This study was conducted in accordance with Declaration of Helsinki and approved by the National Medical Research Register, the regional ethical board in Malaysia. Written informed consent for molecular genotyping was obtained from the subjects prior to blood taking.

## Results

Among the 199 samples investigated, only 17 (8.5%) were positive for β°-thal SEA deletion. Fifteen cases (88.2%) were heterozygous and two cases (11.8%) were compound heterozygous for β°-thal SEA deletion. The results were compared with 182 cases of various heterozygous beta zero deletion, mutation and HPFH types from our database, namely heterozygous β°-thal FIL (*n* = 47), β°-thal 3.5 kb deletion (*n* = 9), Codon 41/42 (-TTCT) (β°) (HBB:c.126_129delCTTT) (*n* = 32), IVS 1-1 (G > T) (β°) (HBB:c.92 + 1G > T) (*n* = 16), HPFH-6 deletion (*n* = 18), Siriraj deletion (*n* = 30) and (δβ)°-thal THAI deletion (*n* = 30). Comparison samples with positive for alpha gene deletions or mutations were excluded from the statistical analysis since all the cases with β°-thal SEA deletion have normal alpha genotype. The demographic data, including age, gender, state, and ethnicity of the β°-thal SEA deletion cases, were analyzed. They were 8 male and 9 female, age ranging from 5 to 48 years old with median ± IQR 16.00 ± 2.00 years. The age of comparison samples were discuss in Table 1. The results showed that β°-thal SEA deletion was most frequently found in Sarawak (*n* = 5; 29.4%), followed by Sabah (*n* = 3; 17.6%) and Selangor (*n* = 3 17.6%). Overall, Chinese patients had the highest number of β°-thal SEA deletion cases (*n* = 10; 58.8%), followed by Bidayuh (*n* = 4; 23.5%) and Sino (*n* = 1; 5.9%). This data demonstrated that β°-thal SEA deletion is common in Malaysia especially in Sarawak, and commonly seen among the Chinese population.

**Table 1:** Hematological parameters and Hb profiles for heterozygotes β°-thalassemia mutation/deletion, HPFH group and (δβ)°-thal THAI deletion.

Comparison groups	Thalassemia mutations	*N*	Age (years)	RBC (10^6^/µl)^a^	Hb (g/dl)^a^	RDW (%)^b^	MCV (fl)^b^	MCH (pg)^b^	Hb A_2_ (%)^b^	Hb F (%)^b^
β°	SEA deletion	15	16.00 ± 2.00^b^	5.48 ± 0.58	13.44 ± 1.45	18.40 ± 3.90	76.00 ± 7.30	24.15 ± 2.38	4.00 ± 1.00	19.50 ± 4.77
	IVS 1-1 mutation	16	35.00 ± 9.70^a^	4.88 ± 1.36	9.74 ± 2.17*	17.45 ± 3.30	62.85 ± 7.20*	19.85 ± 3.05*	5.30 ± 0.45*	1.10 ± 3.85*
	Codon 41/42 mutation	32	33.00 ± 9.00^b^	5.45 ± 1.81	10.62 ± 3.20*	18.00 ± 3.90	62.90 ± 9.00*	19.25 ± 2.18*	5.00 ± 1.35	3.50 ± 14.00*
	FIL deletion	47	33.54 ± 8.83^a^	5.98 ± 0.69	12.26 ± 1.35	15.10 ± 4.10*	63.50 ± 4.18*	20.10 ± 2.44*	6.00 ± 0.58*	2.15 ± 1.15*
	3.5 kb deletion	9	27.71 ± 6.9^a^	5.66 ± 0.87	11.89 ± 1.44	19.55 ± 6.40	67.70 ± 17.45	21.40 ± 3.60*	4.90 ± 3.55	5.60 ± 0.00
^G^γ (^A^γδβ)°	HPFH-6 deletion	18	16.00 ± 9.00^b^	5.59 ± 0.86	13.53 ± 1.63	15.45 ± 3.10*	76.85 ± 5.53	24.80 ± 1.93	2.40 ± 0.25*	22.90 ± 3.43*
	Siriraj deletion	30	16.00 ± 1.00^b^	5.38 ± 0.47	13.20 ± 1.46	14.75 ± 1.80*	75.80 ± 6.13	24.35 ± 1.85	2.40 ± 0.65*	19.60 ± 5.97
(δβ)°	THAI deletion	30	16.00 ± 6.00^b^	5.56 ± 0.68	12.68 ± 1.40	19.45 ± 3.60	71.95 ± 7.73*	23.30 ± 2.38	2.40 ± 0.32*	16.75 ± 5.33*

^a^ Normal distribution data and data presented as mean ± SD using one-way ANOVA and *post hoc* analysis.

^b^ Non-normal distribution data and data presented as median ± IQR using Kruskal Wallis Test; and Mann Whitney Test.

* *p* value < 0.05 compared to β°-SEA deletion group.

Table 1 summarized the haematological parameters and hemoglobin (Hb) profiles of heterozygous β°-thalassemia group, HPFH deletion and (δβ)°-thal THAI deletion. The haematological parameters and Hb profiles of heterozygous β°-thal SEA deletion were significantly difference, except for RBC within the β°-thalassemia group. The mean Hb of heterozygotes β°-thal SEA deletion (13.44 ± 1.45 g/dl) was significantly higher than heterozygotes β°-thalassemia mutations of IVS 1-1 (G > T) (β°) (HBB:c.92 + 1G > T) and Codon 41/42 (-TTCT) (β°) (HBB:c.126_129delCTTT) (*post hoc* test, *p* < 0.05). Even though the mean Hb of heterozygote β°-thal SEA deletion was within the normal range, it was associated with hypochromic microcytic red cells. The median RDW of heterozygotes β°-thal SEA deletion showed a significant different from β°-thal Filipino deletion, HPFH-6 deletion and ^G^γ(^A^γδβ)°-thal Siriraj deletion (Mann Whitney test, *p* < 0.05). The unusual haematological parameters of heterozygous β°-thal SEA deletion, were slightly low MCV (76.00 ± 7.30 fl) and MCH (24.15 ± 2.38 pg) values as compared to other β°-thalassemia group. The median MCV and MCH for β°-thal SEA deletion was significantly higher than other heterozygotes β°-thalassemia traits (Man Whitney test, *p* < 0.05) and more representative of the HPFH group.

The Hb profile analysis of heterozygotes β°-thal SEA deletion showed that the median Hb A_2_ (4.00 ± 1.00%) was in the range of classical β-thalassemia traits and significantly different from IVS 1-1 (G > T) (β°) (HBB:c.92 + 1G > T) mutation, β°-thal FIL deletion, HPFH-6 deletion, (δβ)°-thal THAI deletion and ^G^γ(^A^γδβ)°-thal Siriraj deletion (Mann Whitney test, *p* < 0.05). The Hb A_2_ level was also lower for β°-thalassemia than for both β°-thal FIL deletion and IVS 1-1 (G > T) (β°) (HBB:c.92 + 1G > T) mutation. Interestingly, the Hb F level of heterozygous β°-thal SEA deletion was significantly higher than for other heterozygous β°-thalassemia mutations (*p* < 0.05) except for β°-thal 3.5 kb deletion, and the Hb F level for β°-thal SEA deletion was in the range of HPFH and δβ groups.

Of the 17 cases of β°-thal SEA deletion, one was found to be compound heterozygous β°-thal SEA deletion/Hb E and one was compound heterozygous β°-thal SEA/β°-thal FIL deletion (Table 2). Interestingly, the compound β°-thal SEA/β°-thal FIL deletion patient presented as mild thalassemia intermedia, at the age of 9 years old with a Hb value of 7.3 g/dl at presentation. This case was initially suspected as being beta-thalassemia major, based on a presumptive diagnosis of the Hb analysis findings (Hb F level of 98.8% and Hb A_2_ of 3.8%). Clinically, the patient had no organomegaly or thalassaemic facies. The proband had never received any transfusion. The other patient, with β°-thal SEA deletion/Hb E, presented as thalassemia trait with a Hb value of 11 g/dl. From these two cases, we concluded that the β°-thal SEA deletion is a mild β° phenotype compared to other forms of β°-thalassemia. The haematological parameters for 17 cases of β°-thal SEA deletion were analyzed and showed in Table 2. The possible coexistence of β-thalassemia mutation was investigated in random five samples of β°-thal SEA deletion by performing the β-globin gene sequencing. They showed no significant compound heterozygosity with other β-globin gene mutations that lead to high Hb F level. To complete the genotypic data, the common α-thalassemia deletions [–α^3.7^, –α^4.2^, –^SEA^, –^FIL^, –^MED^ and –(α)^20.5^] and mutations were excluded using multiplex gap-PCR and ARMS-PCR methods respectively (Supplementary Table S2).

**Table 2:** Hematological parameters and Hb profiles of 17 individuals with β°-thal SEA deletion.

Patient	Age (years)	Ethnicity	Genotype	RBC (10^6^/µl)	Hb (g/dl)	RDW (%)	MCV (fL)	MCH (pg)	Hb A_2__HPLC (%)	Hb F_HPLC (%)
P1	16	Chinese	β^SEA^/β, αα/αα	5.00	13	20.5	75.00	25.00	4.00	20.00
P2	30	Chinese	β^SEA^/β, αα/αα	6.00	13	20.0	71.00	22.00	5.00	19.00
P3	16	Chinese	β^SEA^/β, αα/αα	6.00	15	21.2	76.00	24.00	4.00	17.00
P4	16	Chinese	β^SEA^/β, αα/αα	5.00	12	18.1	77.00	24.00	4.00	22.00
P5	48	Bidayuh	β^SEA^/β, αα/αα	5.00	15	16.4	83.00	27.00	5.00	15.00
P6-1	15	Bidayuh	β^SEA^/β, αα/αα	6.00	14	16.3	76.00	25.00	5.00	18.00
P6-2	14	Bidayuh	β^SEA^/β, αα/αα	5.00	13	16.1	72.00	24.00	5.00	16.00
P6-3	5	Bidayuh	β^SEA^/β, αα/αα	5.00	12	15.8	70.00	23.00	4.00	21.00
P7	16	Chinese	β^SEA^/β, αα/αα	5.00	12	-	78.00	25.00	4.00	24.00
P8*	11	Sino	β^SEA^/β^FIL^, αα/αα	3.28	8	27.8	83.10	22.60	3.80	98.80
P9	33	Chinese	β^SEA^/β, αα/αα	6.12	15	22.1	78.60	24.30	4.80	17.90
P10	15	Chinese	β^SEA^/β, αα/αα	5.18	13	18.4	80.10	25.70	3.90	22.00
P11^#^	33	Malay	β^SEA^/β^E^, αα/αα	4.63	11	17.1	71.90	24.60	42.90	46.10
P12	17	Chinese	β^SEA^/β, αα/αα	4.90	11	20.0	70.40	22.20	4.9**	15.9**
P13	17	Brunei	β^SEA^/β, αα/αα	6.45	14	20.0	67.10	21.70	5.2**	14.10**
P14	16	Chinese	β^SEA^/β, αα/αα	5.86	13	20.2	70.10	22.90	4.40	15.00
P15	12	Chinese	β^SEA^/β, αα/αα	6.08	16	17.9	77.30	26.30	3.50	23.60

RBC, red blood cells; Hb, haemoglobin; MCV, mean cell volume; MCH, mean cell haemoglobin; MCHC, mean cell haemoglobin concentration; RDW, red cell distribution width; CE, capillary electrophoresis; HPLC, high performance liquid chromatography.

* Compound heterozygous β^SEA^/β^FIL^: clinically mild thalassaemia intermediate.

** Based on CE result.

^#^ Compound heterozygous β^SEA^/β^E^: clinically trait.

## Discussion

β°-thal SEA deletion was first reported as 27 kb deletion, including the β-globin gene and LCR 3’ Hypersensitive Sites I (HS-1) regulatory sequence (13, 14). Formerly was also described as Vietnamese HPFH by (7) and SEA-HPFH by (11, 15). In 1994, Dimovski (14) have raised the issues comparing whether this deletion should be recognized as beta zero thalassaemia or HPFH. They have reported few aspects including haematological data and *in vitro* gamma chain compensation are likely towards HPFH group. β°-thal SEA deletion was first reported in five members of two families from Southeast Asia (Vietnam and Cambodia) [14]. Previous studies have established that β°-thal SEA deletion is one of the most common deletions found in Southeast Asian populations (7, 16).

**Figure 2:**
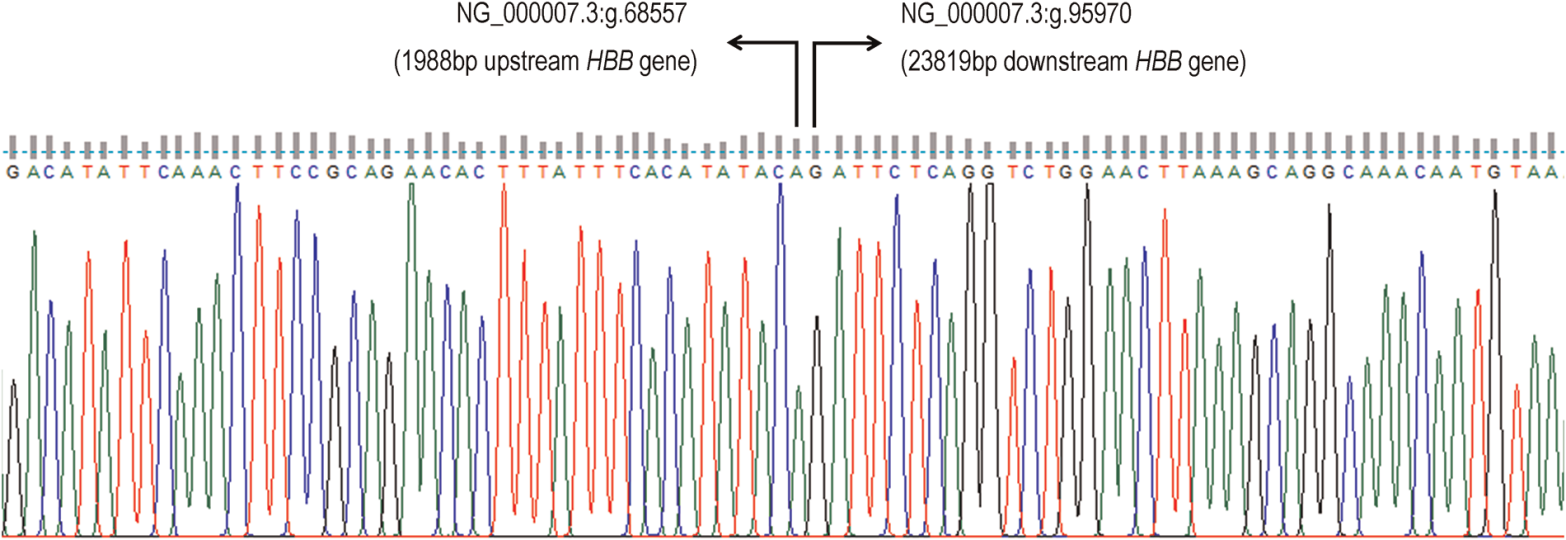
The β°-thal SEA ∼27 kb deletion can be mapped from the 1,987 bp upstream of *HBB* gene to 23,818 bp downstream of β-globin gene (*HBB* gene) (NG_000007.3:g.68558_95969del). Number of nucleotide deleted was 27,412 bp.

Our cohort of cases is larger than the reported cases in China by (15, 16) and supported their findings in which the highest incident of β°-thal SEA deletion occurred among Chinese population. Our study demonstrated that β°-thal SEA deletion is not a rare event and commonly found among Chinese-Malaysian. Based on our data, all the patients presented with mild hypochromic microcytic red blood cells and either normal or subnormal Hb levels. The Hb A_2_ levels were in the classical range of beta thalassemia trait (3.5%–5%), but a pronounced difference from β°-thalassemia trait was in the Hb F level, which are consistent with previous report (16). Based on the statistical analysis comparing the percentage of Hb F level according to their molecular comparison groups, we think that the level of Hb F represents the molecular effect rather than influenced by the physiological raised of HbF in young children, (as the statistical analysis exclusively involved samples from participants more than 14 years old and did not include any pregnancy individuals). Hence we think that the level of HbF is unique and representative of β°-thal SEA deletion. Molecular analysis by gap-PCR and β-globin gene sequencing confirmed the molecular finding of β°-thal SEA deletion, (Figure 2) and similar sequencing data has been reported by (15). We thus confirmed that β°-thal SEA deletion is similar to what was originally reported by Dimovski et al. (7, 11, 13, 15). Other than haematological parameters, the unique feature of heterozygous β°-thal SEA deletion is the phenotype, which is suggestive of the HPFH condition, as observed in two of our cases—one case was a compound heterozygous of β°-thal SEA deletion/Hb E, which was asymptomatic, unlike compound heterozygous of Hb E/β°-thalassemia. The other case was compound heterozygous β°-thal SEA/β°-thal FIL deletion, which presented with mild symptom of an intermediate phenotype with an Hb value of 7.3–9 g/dl (Table 2) and no history of transfusion. The proband was diagnosed at the age of 9 years old after noted pallor with history of acute tonsillitis. The Hb level at diagnosis was 7.3 g/dl. She is currently 17 years old, never had any transfusion till the age of 14 years old at least before loss from follow up. No organomegaly and no thalassemic facies were documented. Our findings is in accordance with a study by (15) where two patients who were compound heterozygous of SEA-HPFH with β°-mutation presented as mild intermediate phenotype with Hb ranges from 10.4 to 11.2 g/dl.

This report provides valuable information for better understanding of the haematological data and prediction of clinical phenotype based on analysis of the breakpoint region (Figure 1). The data of the patients described in this study supports the idea that unusually high Hb A_2_ levels are unique to deletions that remove the 5’ part of β-globin gene region and points to the importance of the 3’ junction sequences for the regulation of Hb F levels in patients with deletion defects of the β-globin gene cluster (4). The elevated Hb A_2_ level in β°-thal SEA deletion that not typically seen in other HPFH type, could be explained by the binding of an intact *HBD* promoter to the transcription factors in place of the deleted β-globin gene promoter (6, 13). Mutations that removed 5’ region of the β-globin gene have unusually high Hb A_2_ and variable Hb F levels. Increased expression of the δ and γ-globin genes in cis is thought to result from enhanced interactions between the LCR and the δ and γ-globin gene promoters as a result of the deletion (10).

Also described as SEA-HPFH (10), β°-thal SEA deletion had higher γ-chain synthesis, which could be due to the removal of the 3’ HS-1 sequence, which has been suggested as a facilitator of the downregulation of 5’ LCR-γ constructs in adult erythroid tissues (7). Compared with β°-thal FIL deletion, which involves a larger deletion area, gene mapping analyzed data by (4), indicated the deletion removes a region of more than 105 Kb, including 3’ HS-1 and the entire β-globin gene (Figure 1). Both β°-thal FIL and β°-thal SEA deletions involve the deletion of a β-globin gene promoter region with different deletion size and 3’ breakpoint regions. The 3’ breakpoints of both deletions involve HS-1 region, with the former deletion involving a larger deletion size than the latter. An earlier study described how the function of the HS-1 region at the 3’ breakpoint of the deletion contains functionally important sequences and that the juxtaposition of these sequences with the γ-globin genes is a significant factor in the increased Hb F level (6). The fact that the 3’ HS-1 sequence is also deleted by β°-thal FIL deletion, but without high Hb F levels like β°-thal SEA deletion, demonstrates that this element by itself may not be sufficient to have a silencing effect on γ-globin gene expression. A possible explanation for these findings may be the preserved of γ-gene-specific enhancer element found close to the 3’ HS-1 breakpoint of the β°-thal SEA deletion, as compared to β°-thal FIL deletion (13), thus explaining the difference in mean Hb F level in β°-thal FIL deletion (much lower level than the Hb F levels for β°-thal SEA deletion) (Table 1, Figure 1).

Beyond this, a new Caucasian HPFH deletion has been discovered with β-thalassemia-like Hb A_2_ levels and involving a 27,825 bp deletion with a 25 bp insertion, similar to β°-thal SEA deletion. The haematological indices and red blood cell parameters are similar to β°-thal SEA deletion, with Hb F levels greater than 20% (1). Neither of the deletions remove β-δ intergenic region located between ^A^γ and δ-globin genes recently described by (14) to be common in all HPFH deletions. The pseudo β-δ intergenic region has an important role as silencing element for the γ-globin gene. This might suggest that a mechanism other than *BCL11A* gene expression could account for the silencing of the γ-globin genes during the first months of life (8). Another proposed model could explain the possible causes of high Hb F level in (δβ)° and HPFH deletions (14). The enhancer sequence located at the downstream end of the deletions may determine the outcome when strong enhancer elements are juxtaposed closer to the γ-globin gene as a result of the deletions, leading to higher Hb F level. The β°-thal SEA deletion exhibits milder phenotypes than other β°-thalassemia deletions, which may be explained by the beneficial effect of Hb F on red blood cell production and survival and amelioration of the clinical phenotype.

In summary, our study provides comprehensive haematological parameters of β°-thal SEA deletion that possibly can create awareness regarding the important details for understanding β°-thal SEA deletion and comparisons with the β°-thalassemia group, HPFH and δβ group. It is noteworthy that, although the deletion regions involved only the β-globin gene, they presented as a phenotype of HPFH. By comparing those groups, β°-thal SEA deletion better classified under HPFH group rather than beta zero group. Determining deletion breakpoints within the β-globin gene cluster could give a clue towards an understanding of the haematological data. From Malaysian perspective, the study also demonstrates that β°-thal SEA deletion is not only common among Chinese ethnicity, as reported by (11, 15, 16) but also discovered among Malays and Indigenous ethnicity in Sarawak namely Sino and Kadazan. A study of the specific mutations, especially deletional-type of β-thalassemia, would provide valuable understanding of the effect of those mutations on the activity of globin genes (δ and γ genes). As described by (7, 10, 11, 15) we affirm to classifying this deletion as SEA-HPFH, considering the phenotype features rather than the exact genetic lesion, as this issue will affect the counselling of affected individuals. The term of beta zero (β°) that we are currently used is misnomer and will lead to confusion especially to clinician involved in genetic counselling of the patients. Determining the breakpoints within the β-globin gene cluster would offer towards an understanding of the haematological data and the expected clinical phenotype.

Other than that, this finding is important and impacts the plans for molecular tests, especially in a country with limited resources. Cases with a classical Hb A_2_ of β°-thalassemia, with a profoundly high Hb F level and slightly low or normal Hb levels would fulfil the criteria for β°-thal SEA deletion detectable by conventional GAP-PCR, leading to significant reduction in additional testing such as β-globin gene sequencing and MLPA.

## Data Availability

The original contributions presented in the study are included in the article/**Supplementary Material**, further inquiries can be directed to the corresponding author/s.

## References

[1] Pissard S Raclin V Lacan P Garcia C Aguilar-Martinez P Francina A Characterization of three new deletions in the β-globin gene cluster during a screening survey in two French urban areas. Clin Chim Acta. (2013) 415:35–40. 10.1016/j.cca.2012.08.030 22981786

[2] Harteveld CL Voskamp A Phylipsen M Akkermans N Den Dunnen JT White SJ Nine unknown rearrangements in 16p13.3 and 11p15.4 causing alpha- and beta-thalassaemia characterised by high resolution multiplex ligation-dependent probe amplification. J Med Genet. (2005) 42(12):922–31. 10.1136/jmg.2005.033597 15894596 PMC1735959

[3] Tritipsombut J Phylipsen M Viprakasit V Chalaow N Sanchaisuriya K Giordano PC A single-tube multiplex gap-polymerase chain reaction for the detection of eight β-globin gene cluster deletions common in Southeast Asia. Hemoglobin. (2012) 36(6):571–80. 10.3109/03630269.2012.747441 23181748

[4] Thein SL. The molecular basis of β-thalassemia. Cold Spring Harb Perspect Med. (2013) 3(5):1–24. 10.1101/cshperspect.a011700 PMC3633182 23637309

[5] Giardine BM, Joly P, Pissard S, Wajcman H, Chui DHK, et al. Clinically relevant updates of the HbVar database of human hemoglobin variants and thalassemia mutations. *Nucleic Acids Res*. (2021), 49:D1192–6. 10.1093/nar/gkaa959 PMC7778921 33125055

[6] Dimovski AJ Baysal E Efremov DG Prior JF Raven JL Efremov GD A large beta thalassemia deletion in family of Indonesian-malay descent. Hemoglobin. (1996) 53(9):1689–99. 10.3109/03630269609005842 8936464

[7] Motum PI Hamilton TJ Lindeman R Le H Trent RJ. Molecular characterisation of vietnamese HPFH. Hum Mutat. (1993) 1993(2):179–84. 10.1002/humu.1380020305 7689901

[8] Bain BJ. (editor). The α, β, δ and γ thalassaemias and related conditions. In: *Haemoglobinopathy diagnosis*. Blackwell Publishing Ltd. (2006) p. 63–138. 10.1002/9780470988787.ch3

[9] Ryan K Bain BJ Worthington D James J Plews D Mason A Significant haemoglobinopathies: guidelines for screening and diagnosis. Br J Haematol. (2010) 149(1):35–49. 10.1111/j.1365-2141.2009.08054.x 20067565

[10] Changsri K Akkarapathumwong V Jamsai D Winichagoon P Fucharoen S. Molecular mechanism of high hemoglobin F production in Southeast Asian-type hereditary persistence of fetal hemoglobin. Int J Hematol. (2006) 83(3):229–37. 10.1532/IJH97.E0509 16720553

[11] Xu X-M Li Z-Q Liu Z-Y Zhong X-L Zhao Y-Z Mo Q-H. Molecular characterization and PCR detection of a deletional HPFH: application to rapid prenatal diagnosis for compound heterozygotes of this defect with β-thalassemia in a Chinese family. J Hematol. (2000) 65:183–8. 10.1002/1096-8652(200011)65:3<183::AID-AJH1>3.0.CO;2-R 11074532

[12] Syahzuwan H Rahimah A Zubaidah Z Syahzuwan H Rahimah A Zubaidah Z. Detection of β -globin gene mutations among β -thalassaemia carriers and patients in Malaysia: application of multiplex amplification refractory mutation system – polymerase chain reaction. Malays J Med Sci. (2013) 20(1):13–20. PMID: 23613656 PMC3629881

[13] Thong MK Tan JAMA Tan KL Yap SF. Characterisation of β-globin gene mutations in Malaysian children: a strategy for the control of β-thalassaemia in a developing country. J Trop Pediatr. (2005) 51(6):328–33. 10.1093/tropej/fmi052 15967770

[14] Dimovski AJ Divoky V Adekile AD Baysal E Wilson JB Prior JF A novel deletion of ∼27 kb including the β-globin gene and the locus control region 3’HS-1 regulatory sequence: β°-thalassemia or hereditary persistence of fetal hemoglobin? Blood. (1994) 83(3):822–7. 10.1182/blood.V83.3.822.822 7507736

[15] He S Wei Y Lin L Chen Q Yi S Zuo Y The prevalence and molecular characterization of (δβ)0-thalassemia and hereditary persistence of fetal hemoglobin in the Chinese zhuang population. J Clin Lab Anal. (2018) 32(3):1–6. 10.1002/jcla.22304 PMC5888142 28763119

[16] Cai WJ Li J Xie XM Li DZ. Screening for common β-globin gene cluster deletions in Chinese individuals with increased hemoglobin F. Int J Lab Hematol. (2015) 37(6):752–7. 10.1111/ijlh.12401 26179971

