# Supplementary material for: Molecular and hematological studies in a cohort of beta zero South East Asia deletion (β°-thal SEA) from Malaysian perspective
Source: Front Pediatr. 2022 Nov 30;10:974496. doi: 10.3389/fped.2022.974496 (PMC9748471; doi:10.3389/fped.2022.974496)
Supplement: Supplementary file 1 [file Table1.docx]

| ID | **Age** | **Ethnicity** | **Gender** | **Phenotype** | **Genotype alpha and beta** | **Test done** | **RBC (10^6^/µL)** | **Hb  (g/dL)** | **MCV  (fL)** | **MCH (pg)** | **HPLC** | | **CE** | |
| --- | --- | --- | --- | --- | --- | --- | --- | --- | --- | --- | --- | --- | --- | --- |
|  |  |  |  |  |  |  |  |  |  |  | **Hb A_2_** | **Hb F  (%)** | **Hb A_2_ (%)** | **Hb F  (%)** |
| P1 | 16 | Chinese | Male | Trait | β^SEA^ /β, αα/αα | α-MGAP, α-MARMS, β-MGAP, *β-sequencing | 5.00 | 13 | 75.00 | 25.00 | 4.00 | 20.00 | - | - |
| P2 | 30 | Chinese | Female | Trait | β^SEA^ /β, αα/αα | α-MGAP, α-MARMS, β-MGAP, β-MARMS | 6.00 | 13 | 71.00 | 22.00 | 5.00 | 19.00 | - | - |
| P3 | 16 | Chinese | Male | Trait | β^SEA^ /β, αα/αα | α-MGAP, α-MARMS, β-MGAP, *β-sequencing | 6.00 | 15 | 76.00 | 24.00 | 4.00 | 17.00 | 4.00 | 19.00 |
| P4 | 16 | Chinese | Female | Trait | β^SEA^ /β, αα/αα | α-MGAP, α-MARMS, β-MGAP | 5.00 | 12 | 77.00 | 24.00 | 4.00 | 22.00 | 4.00 | 22.00 |
| P5 | 48 | Bidayuh | Male | Trait | β^SEA^ /β, αα/αα | α-MGAP, α-MARMS, β-MGAP, β-MARMS | 5.00 | 15 | 83.00 | 27.00 | 5.00 | 15.00 | - | - |
| P6-1 | 15 | Bidayuh | Male | Trait | β^SEA^ /β, αα/αα | α-MGAP, α-MARMS, β-MGAP, β-MARMS | 6.00 | 14 | 76.00 | 25.00 | 5.00 | 18.00 | - | - |
| P6-2 | 14 | Bidayuh | Female | Trait | β^SEA^ /β, αα/αα | α-MGAP, α-MARMS, β-MGAP, β-MARMS | 5.00 | 13 | 72.00 | 24.00 | 5.00 | 16.00 | - | - |
| P6-3 | 5 | Bidayuh | Female | Trait | β^SEA^ /β, αα/αα | α-MGAP, α-MARMS, β-MGAP | 5.00 | 12 | 70.00 | 23.00 | 4.00 | 21.00 | - | - |
| P7 | 16 | Chinese | Female | Trait | β^SEA^ /β, αα/αα | β-MGAP, *β-sequencing | 5.00 | 12 | 78.00 | 25.00 | 4.00 | 24.00 | 3.00 | 28.00 |
| P8 | 11 | Sino | Female | Intermedia | β^SEA^ /β^FIL^, αα/αα | α-MGAP, α-MARMS, β-MGAP, β-MARMS | 3.28 | 8 | 83.10 | 22.60 | 3.80 | 98.80 | 2.40 | 97.60 |
| P9 | 33 | Chinese | Male | Trait | β^SEA^/β, αα/αα | α-MGAP, α-MARMS, β-MGAP, β-MARMS | 6.12 | 15 | 78.60 | 24.30 | 4.80 | 17.90 | 4.70 | 19.70 |
| P10 | 15 | Chinese | Female | Trait | β^SEA^/β, αα/αα | α-MGAP, α-MARMS, β-MGAP, *β-sequencing | 5.18 | 13 | 80.10 | 25.70 | 3.90 | 22.00 | - | - |
| P11 | 33 | Malay | Female | Trait | β^SEA^/β^E^, αα/αα | α-MGAP, α-MARMS, β-MGAP, β-MARMS, β-MARMS HbE | 4.63 | 11 | 71.90 | 24.60 | 42.90 | 46.10 | - | - |
| P12 | 17 | Chinese | Female | Trait | β^SEA^/β, αα/αα | α-MGAP, α-MARMS, β-MGAP | 4.90 | 11 | 70.40 | 22.20 | - | - | 4.90 | 15.90 |
| P13 | 17 | Brunei | Male | Trait | β^SEA^/β, αα/αα | α-MGAP, α-MARMS, β-MGAP | 6.45 | 14 | 67.10 | 21.70 | - | - | 5.20 | 14.10 |
| P14 | 16 | Chinese | Female | Trait | β^SEA^/β, αα/αα | β-MGAP | 5.86 | 13 | 70.10 | 22.90 | 4.40 | 15.00 | 4.30 | 15.80 |
| P15 | 17 | Chinese | Male | Trait | β^SEA^/β, αα/αα | α-MGAP, α-MARMS, β-MGAP, *β-sequencing | 6.08 | 16 | 77.30 | 26.30 | 3.50 | 23.60 | - | - |

Abbreviation: RBC, red blood cells; Hb, haemoglobin; MCV, mean cell volume; MCH, mean cell haemoglobin; MCHC, mean cell haemoglobin concentration; RDW, red cell distribution width

CE, Capillary electrophoresis; HPLC, High Performance Liquid Chromatography

multiplex gap-PCR and multiplex ARMS-PCR for HBA1 and HBA2 gene were performed in all 17 cases. *Five cases were randomly selected for *HBB* gene sequencing analysis to exclude compound heterozygous with other beta thalassaemia variant
